# Human Neutrophil Cytoskeletal Dynamics and Contractility Actively Contribute to Trans-Endothelial Migration

**DOI:** 10.1371/journal.pone.0061377

**Published:** 2013-04-23

**Authors:** Kimberly M. Stroka, Heather N. Hayenga, Helim Aranda-Espinoza

**Affiliations:** Fischell Department of Bioengineering, University of Maryland, College Park, Maryland, United States of America; University of Bristol, United Kingdom

## Abstract

Transmigration through the endothelium is a key step in the immune response. In our recent work, the mechanical properties of the subendothelial matrix and biophysical state of the endothelium have been identified as key modulators of leukocyte trans-endothelial migration. Here, we demonstrated that neutrophil contractile forces and cytoskeletal dynamics also play an active biophysical role during transmigration through endothelial cell-cell junctions. Using our previously-established model for leukocyte transmigration, we first discovered that >93% of human neutrophils preferentially exploit the paracellular mode of transmigration in our *in vitro* model, and that is independent of subendothelial matrix stiffness. We demonstrated that inhibition of actin polymerization or depolymerization completely blocks transmigration, thus establishing a critical role for neutrophil actin dynamics in transmigration. Next, inhibition of neutrophil myosin II-mediated contractile forces renders 44% of neutrophils incapable of retracting their trailing edge under the endothelium for several minutes after the majority of the neutrophil transmigrates. Meanwhile, inhibition of neutrophil contractile forces or stabilization of microtubules doubles the time to complete transmigration for the first neutrophils to cross the endothelium. Notably, the time to complete transmigration is significantly reduced for subsequent neutrophils that cross through the same path as a previous neutrophil and is less dependent on neutrophil contractile forces and microtubule dynamics. These results suggest that the first neutrophil induces a gap in endothelial cell-cell adhesions, which “opens the door” in the endothelium and facilitates transmigration of subsequent neutrophils through the same hole. Collectively, this work demonstrates that neutrophils play an active biophysical role during the transmigration step of the immune response.

## Introduction

Trans-endothelial migration is a key step in the inflammatory response and is the means by which leukocytes exit the bloodstream and enter an infected tissue. During transmigration, biological signaling cascades are activated in both the leukocytes [1], [2] and the endothelial cells (ECs) [1], [3], [4], [5], [6] through which they migrate. Leukocytes readily exploit two different transmigration pathways in which they either squeeze through endothelial cell-cell junctions (paracellular route) or directly through ECs (transcellular route). In general, the preferential pathway taken depends *in vitro* on the type of ECs or *in vivo* by vascular location [7], [8], [9], [10], [11], [12], [13], and the biological signaling varies accordingly. For example, as a leukocyte migrates between EC borders, junctional proteins such as vascular endothelial cadherin (VE-cadherin) are laterally displaced to create an intercellular gap [10]. Meanwhile, transcellular transmigration is initiated through formation of a “transmigratory cup” enriched with intercellular adhesion molecule-1 (ICAM-1) and vascular cell adhesion molecule-1 (VCAM-1), along with actin-containing microvilli [8], [14]. A thorough review on the molecular mechanisms of paracellular and transcellular transmigration can be found elsewhere [15]. In both cases, however, the endothelium prepares itself by rearranging key biological proteins to enable transmigration.

At the same time, transmigration is also a biophysical event [16] that involves exertion of physical forces [1], [17], [18]. Specifically, the biophysical properties of the endothelium have recently been implicated in barrier function and transmigration [1], [6], [19], [20]. In addition, we have discovered that the subendothelial matrix is a key mediator of transmigration through the endothelium [1], [6]. In particular, stiffer subendothelial matrices support enhanced EC contractile forces and formation of intercellular gaps that promote increased neutrophil transmigration. We established that EC myosin light chain kinase (MLCK) is a key molecule driving the mechanosensitive response of transmigration through both tumor necrosis factor-alpha (TNF-α)- and oxidized low-density lipoprotein (oxLDL)-treated endothelium [1], [6]. Thus, the endothelium plays an important biophysical role in neutrophil transmigration. We have also discovered that neutrophils are mechanosensitive, as their migration speed is biphasic with matrix stiffness [21], suggesting that their role in transmigration may involve their own biophysical machinery.

Using the technique of traction force microscopy, human neutrophils have been shown to produce significant and detectable traction forces on flexible polyacrylamide gels of similar stiffness to the native endothelium [22], [23]. Notably, these forces are localized to the uropod at the rear of the cell [23]. In addition, engineered substrates consisting of extracellular matrix-coated micropillars which deflect as cells exert traction have been used to measure force production by ECs and leukocytes during transmigration [17], [18]. In these assays, forces are significantly enhanced during monocyte [17] or neutrophil [18] transmigration; these results further demonstrate that transmigration is a biophysical event. However, little is known about the molecular machinery active in the neutrophils as they transmigrate. Therefore, in the current work, we hypothesized that neutrophil contractile forces and cytoskeletal dynamics play an active biophysical role during transmigration through endothelial cell-cell junctions.

In order to explore the biophysical role of neutrophils in transmigration, we utilized a combination of engineered subendothelial matrices of physiologically relevant stiffness, live cell timelapse fluorescence and phase contrast microscopy, pharmacological drug treatments, and quantitative analysis. Our results here demonstrate that transmigration involves a coordinated biological and biophysical effort that implicates neutrophil actin and microtubule dynamics, as well as MLCK- and myosin II-mediated contractility. Collectively, this work demonstrates that neutrophils play an active biophysical role in the transmigration step of the immune response, a knowledge that could be exploited in development of drug delivery strategies, immune disorder therapeutics, or cardiovascular disease treatments.

## Materials and Methods

### Substrate preparation

Thin polyacrylamide gels were prepared on 22×22 mm coverslips (No. 1.5, Fisher Scientific, Pittsburgh, PA) by the method first described by Pelham and Wang [24] and as previously described in our work [1], [21], [25]. In this work, concentrations of 15% acrylamide+1.2% bis (280 kPa), 8% acrylamide+0.07% bis (5 kPa), and 3% acrylamide+0.1% bis (0.87 kPa) were used. Gels were coated with 0.1 mg/mL fibronectin (Sigma Aldrich, St. Loius, MO) as previously described [1], [21], [25]. Gel mechanical properties were characterized using atomic force microscopy and dynamic mechanical analysis, and surface-bound fibronectin was characterized using fluorescently-tagged anti-fibronectin antibodies and fluorescence microscopy, as previously described [1], [21], [25].

### Endothelial cell culture

Human umbilical vein ECs (HUVECs; Lifeline Cell Technology, Walkersville, MD) were cultured as previously described [1], [26]. Cells (passages 2–5; 4×10^5^ total) were plated onto fibronectin-coated polyacrylamide gels and formed monolayers within 2 days. Monolayers were then incubated with 25 ng/mL TNF-α (Fisher Scientific) for 24 hours and washed with PBS prior to adding neutrophils.

### Adenovirus transfection

HUVECs were transfected with VE-cadherin-GFP (VEcadGFP) using an adenovirus (AdV), which was received as a gift from Dr. William Luscinskas (Harvard Medical School). Construction of the VEcadGFP plasmid and transference to an adenovirus expression vector were previously described in work from Dr. Luscinskas's lab [10]. HUVECs were plated onto fibronectin-coated polyacrylamide gels and given 1–2 hours to spread. After attachment and spreading, 3 µL of AdV-VEcadGFP were added to the cells with 2 mL media. HUVECs were incubated for 1–2 days, and finally 25 ng/mL TNF-α was added for an additional 24 hours. HUVECs were washed with PBS prior to adding neutrophils.

### Neutrophil isolation and treatments

Neutrophils were isolated from human blood using centrifugation through Polymorphprep media (Accurate Chemical, Westbury, NY), as previously described in detail [21]. To interfere with neutrophil cytoskeletal dynamics and contraction, neutrophils were incubated with various pharmacological drugs in suspension. Drugs included DMSO (Fisher Scientific) as the vehicle control, blebbistatin (15 µM, 5 minutes; Sigma Aldrich) to inhibit myosin II, ML-7 (15 µM, 8 minutes; Sigma Aldrich) to inhibit MLCK, nocodazole (5 µM, 5 minutes; Sigma Aldrich) to inhibit microtubule polymerization, taxol (1 µM, 30 minutes; Sigma Aldrich) to stabilize microtubules, jasplakinolide (1 µM, 5 minutes; Calbiochem) to stabilize and polymerize actin, and latrunculin-A (1 µM, 30 minutes; Sigma Aldrich) to disrupt actin. After treatment in suspension, neutrophils were centrifuged and washed twice in HBSS to ensure no residual drug was left in the cell suspension.

### Ethics statement

All human blood donors provided written informed consent, and all methods were approved by the University of Maryland Institutional Review Board.

### Transmigration assays

Neutrophils (1×10^6^ total) were plated onto TNF-α-activated HUVECS immediately after drug treatment. Within about 30–60 seconds, neutrophils gravitated to the endothelium, and phase contrast images were captured every 5 seconds for 30 minutes by time-lapse microscopy. In experiments where the endothelium was infected with AdV-VEcadGFP, fluorescence images were also captured simultaneously. Microscopy was completed at 37°C, 5% CO_2_ and 55% humidity using an inverted microscope (Olympus IX71, Center Valley, PA). Images were captured with either a QImaging Retiga-SRV charge-coupled device (CCD) digital camera (QImaging Corporation, Surrey, British Columbia, Canada) using IPLab software (Becton, Dickinson and Company, Franklin Lakes, NJ), or with a a QImaging Rolera-MGi CCD digital camera (QImaging Corporation) using Slidebook software (version 4.2.0.9; Intelligent Imaging Innovations, Denver, CO).

### Quantitative analysis of transmigration

Fraction of transmigration was quantified by dividing the number of cells that transmigrated by the total number of neutrophils in the first frame of the movie. Cells that entered or exited the frame of view were not analyzed. Transmigration initiation time was taken as the frame just prior to the one where the first protrusion was seen beneath the endothelium, while complete transmigration occurred when the entire neutrophil was phase-darkened beneath the endothelium. Time to transmigrate was calculated as the difference between the time of initiation and complete transmigration. For the case where neutrophils attempted transmigration more than once (and migrated some distance in between attempts), the initiation time was taken only for the attempt in which the neutrophil successfully transmigrated. In the experiments where the endothelium was infected with AdV-VEcadGFP, neutrophils that protruded beneath the endothelium at a cell-cell junction were considered to transmigrate via the paracellular route (bicellular if at the junction between two cells, or tricellular if at the junction between three cells), while neutrophils that did not protrude at a cell-cell junction were considered to transmigrate via the transcellular route.

### Immunostaining

To confirm efficacy of drug treatments, neutrophils were treated with various drugs at the previously-specified concentrations and incubation times (Fig. S1). Glass-bottom dishes (MatTek, Ashland, MA) were coated with 0.1 mg/mL fibronectin (Sigma Aldrich) for 2 hours at room temperature. Neutrophils (1×10^4^ cells) were added to each dish and activated with 40 ng/mL fMLF (MP Biomedicals, Santa Ana, CA) for 1 hour at 37°C. Neutrophils were fixed for 20 minutes in 3% glutaraldehyde (Ted Pella, Redding, CA) before drug treatment, immediately after drug treatment, or 30 minutes after drug washout. After washing with PBS, neutrophils were permeabilized in 1% Triton-X 100 (VWR International, West Chester, PA) for 5 minutes, and blocked for nonspecific binding in 10% bovine serum albumin (BSA; Sigma Aldrich) for 30 minutes at room temperature. Neutrophils were incubated with one of the following primary antibodies, each at 1∶100 dilution, for 1 hour at room temperature: myosin IIA (10 µg/mL; ab24762-50, Abcam, Cambridge, MA), myosin light chain kinase (MLCK) (ab76092, Abcam), or α-tubulin (10 µg/mL; ab7291, Abcam, Cambridge, MA). To visualize actin, cells were stained with Phalloidin-TRITC (1 µM; Sigma Aldrich). After washing two times with PBS, samples were incubated with one of the followig secondary antibodies at a 1∶100 dilution for 1 hour at room temperature: Alexa Fluor 561 goat anti-mouse IgG (2 µg/mL; A-11020, Life Technologies, Grand Island, NY) or Alexa Fluor 594 goat anti-rabbit IgG (2 µg/mL; A-11037, Life Technologies). Nuclei were stained with 4 µg/mL Hoechst dye (33342, Invitrogen, Grand Island, NY) for 10 minutes at room temperature. After staining, neutrophils were washed two times with PBS. Images were captured using the same exposure time and settings for each set of treatments (i.e. pre-drug, post-drug, and washout) on an inverted Olympus microscope as described above.

### Statistical analysis

All experiments were repeated at least three times independently. Data are reported as mean ± standard error (SEM) unless otherwise noted. Statistical significance was determined using analysis of variance between groups, followed by Tukey's honestly significant difference criterion, or using a Student's t-test for pairs of data. A P-value of less than 0.05 was considered statistically significant.

## Results

### Neutrophils preferentially take the paracellular route of transmigration

**Figure 1:**
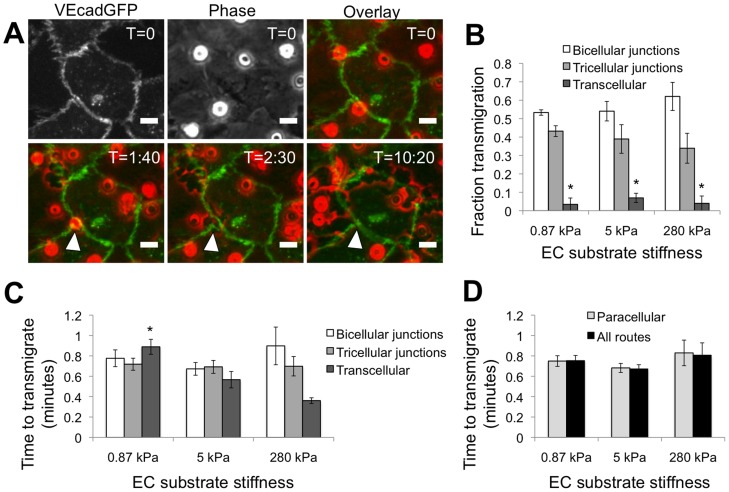
Location of transmigration. (A) Example of a neutrophil transmigrating at a tricellular junction of endothelial cells. The images were color-merged in ImageJ (VE-cadherin-GFP, green; phase contrast, red) to observe the location of transmigration in relation to endothelial cell borders. The white arrowhead points to the cell of interest that is transmigrating at the intersection of three endothelial cells. The time after plating neutrophils (in minutes∶seconds format) onto the endothelium is indicated in the top right corner of each image. Scale bar is 10 µm in all images. (B) Fraction of transmigrated cells (not total cells) that transmigrate via the bicellular junctions, tricellular junctions, or transcellular route, as a function of subendothelial matrix stiffness. Bars represent mean ± SEM of 3 independent experiments. * indicates P<0.05 with bicellular and tricellular junction routes on the same substrate stiffness by ANOVA, followed by Tukey's honestly significant difference criterion. There were no statistical differences between substrates for any route. (C) Time to complete transmigration as a function of route and subendothelial matrix stiffness. Bars represent mean ± SEM of pooled data from 3 independent experiments (on 0.87 kPa, N = 45, 37, 3 for bicellular, tricellular, and transcellular pathways, respectively; on 5 kPa, N = 28, 26, 5; on 280 kPa, N = 40, 21, 3). * indicates P<0.05 with 5 kPa and 280 kPa for the transcellular route by ANOVA, followed by Tukey's honestly significant difference criterion. (D) Time to complete transmigration is shown for all cells regardless of route taken, or cells taking the paracellular route (includes bicellular and tricellular junctions), as a function of subendothelial matrix stiffness. There were no statistical differences between any substrates or routes.

As previously noted, neutrophils are capable of transmigrating via the paracellular or transcellular route. Both methods have previously been observed *in vitro* and *in vivo* [7], [8], [9], [10], [11], [12], though it has been suggested that transmigration *in vivo* occurs predominantly through the endothelial cell-cell junctions in certain tissues [13]. Therefore, our first goal was to evaluate the frequency of paracellular versus transcellular transmigration in our system, since it is likely that the biophysical role of neutrophils may vary depending on the route taken. We infected the monolayers of HUVECs with an adenovirus containing AdV-VEcadGFP in order to visualize endothelial cell-cell borders in live cells (Fig. 1A; Movie S1). In agreement with previous reports [10], we observed VE-cadherin-GFP dislocation as neutrophils transmigrated via the paracellular route (Fig. 1A, white arrowhead). Of the neutrophils that transmigrated, more than 93% used the paracellular route on all substrates, indicating that this route was most preferential for transmigration in our assays (Fig. 1B; P<0.05). Further, of the neutrophils that transmigrated via the paracellular route, they equally exploited the bicellular and tricellular routes (as defined in the Materials and Methods section) on all substrates (Fig. 1B). Gaps in VE-cadherin-GFP typically closed within several minutes after the neutrophil completed transmigration (Fig. 1A, white arrowhead; Movie S1).

### Transmigration route does not depend on subendothelial matrix stiffness

Because we have previously identified subendothelial matrix stiffness as an important regulator of neutrophil transmigration [1], we also evaluated the mode of transmigration as a function of subendothelial matrix stiffness. We originally hypothesized that endothelial contractility, which depends on subendothelial matrix stiffness [1], [20], is an important parameter in a neutrophil's decision of what transmigration route to exploit. In agreement with our previous work [1], we found that neutrophil transmigration through AdV-VEcadGFP-infected HUVECs increased with increasing subendothelial matrix stiffness. However, for all modes of transmigration (bicellular, tricellular, transcellular), there was no difference between soft (0.87 kPa), intermediate (5 kPa), and stiff (280 kPa) substrates (Fig. 1B; P>0.05). Therefore, even though subendothelial matrix stiffness affects the overall fraction of neutrophils that transmigrate, it does not govern the route by which neutrophils cross the endothelial barrier.

### Time to complete transmigration does not depend on paracellular mode of transmigration or subendothelial matrix stiffness

In addition to quantifying the fraction of neutrophils that transmigrate for a particular condition, we also measured the mean time required for a neutrophil to completely traverse the endothelium. For each subendothelial matrix stiffness, there was no difference in transmigration time between the bicellular, tricellular, and transcellular routes (Fig. 1C; P>0.05 for all substrates). Meanwhile, for both paracellular routes (bicellular and tricellular), there was no difference in transmigration time between substrates (Fig. 1C; P>0.05). Interestingly, for the transcellular route, transmigration time on the softest substrate (0.87 kPa, where EC contractility is reduced [1], [20]) was larger than on the stiffer substrates (5 and 280 kPa) (Fig. 1C; P<0.05), suggesting that neutrophils can more easily transmigrate through the bodies of ECs in which contractile forces are higher. However, to more fully explore this phenomenon, a system is necessary where a higher percentage of neutrophils utilize the transcellular route, as in the case of human dermal (HDMVEC) or lung (HLMVEC) microvascular endothelial cells [7].

Finally, we found that when we classified the neutrophils more generally, there was no difference in transmigration time between those taking the paracellular route in comparison with all transmigrating cells (P>0.05), and this held for all substrates (Fig. 1D). Importantly, this indicates that in comparing transmigration times, the transcellular route held little weight in comparison with the paracellular routes, since a small fraction of neutrophils utilized the transcellular route (<7% for all substrates). Thus, for the remainder of the experiments discussed below, we chose to focus on the intermediate subendothelial matrix stiffness (5 kPa), which is in the “healthy” range physiologically [27], [28], [29].

### Inhibition of myosin II or actin polymerization/depolymerization reduces transmigration

To evaluate the biophysical role of neutrophils in transmigration through the vascular endothelium, we treated neutrophils with various pharmacological drugs to inhibit contractile forces (blebbistatin, ML-7), depolymerize or stabilize microtubules (nocodazole, taxol), or depolymerize or stabilize actin (latruculin-A, jasplakinolide). We first verified stabilization or disruption of target proteins via fluorescent staining before drug treatment, after drug treatment, and after washout (Fig. S1). Collectively, we observed that immediately after treatment, or even 30 minutes after washout, each drug's effect on microtubule structure, myosin IIA, MLCK, or actin organization was still apparent (Fig. S1).

**Figure 2:**
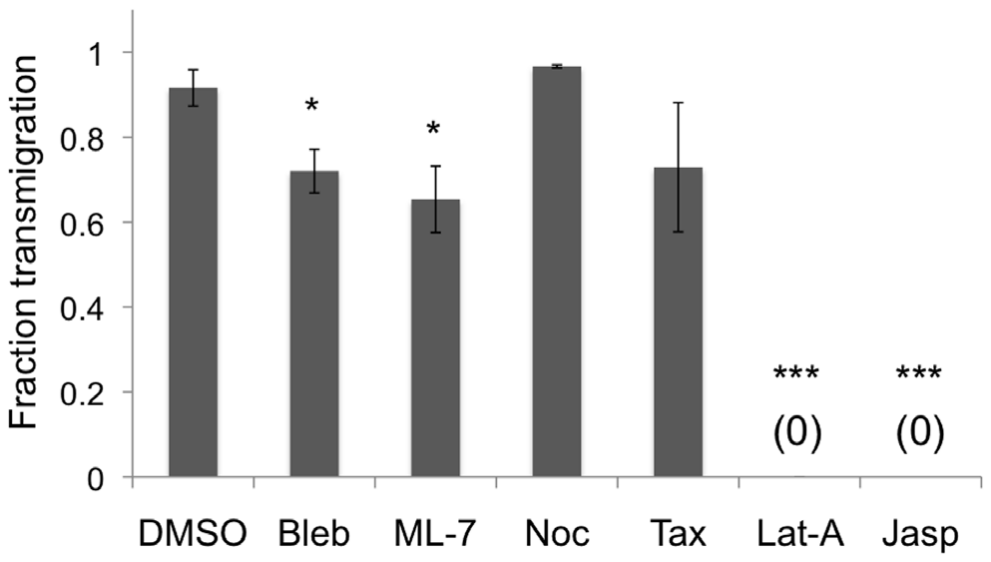
Fraction of neutrophils that transmigrate. Neutrophils were pretreated with DMSO (vehicle control), blebbistatin (“Bleb”; to inhibit myosin II), ML-7 (to inhibit myosin light chain kinase), nocodazole (“Noc”; to inhibit microtubule polymerization), taxol (“Tax”; to stabilize microtubules), latrunculin-A (“Lat-A”; to inhibit actin polymerization) or jasplakinolide (“Jasp”; to inhibit actin depolymerization) and plated onto a TNF-α-activated HUVEC monolayer on a 5 kPa polyacrylamide gel. Bars represent mean ± SEM of 3–4 independent experiments. * indicates P<0.05, and *** indicates P<0.001 with DMSO control using a t-test.

**Figure 3:**
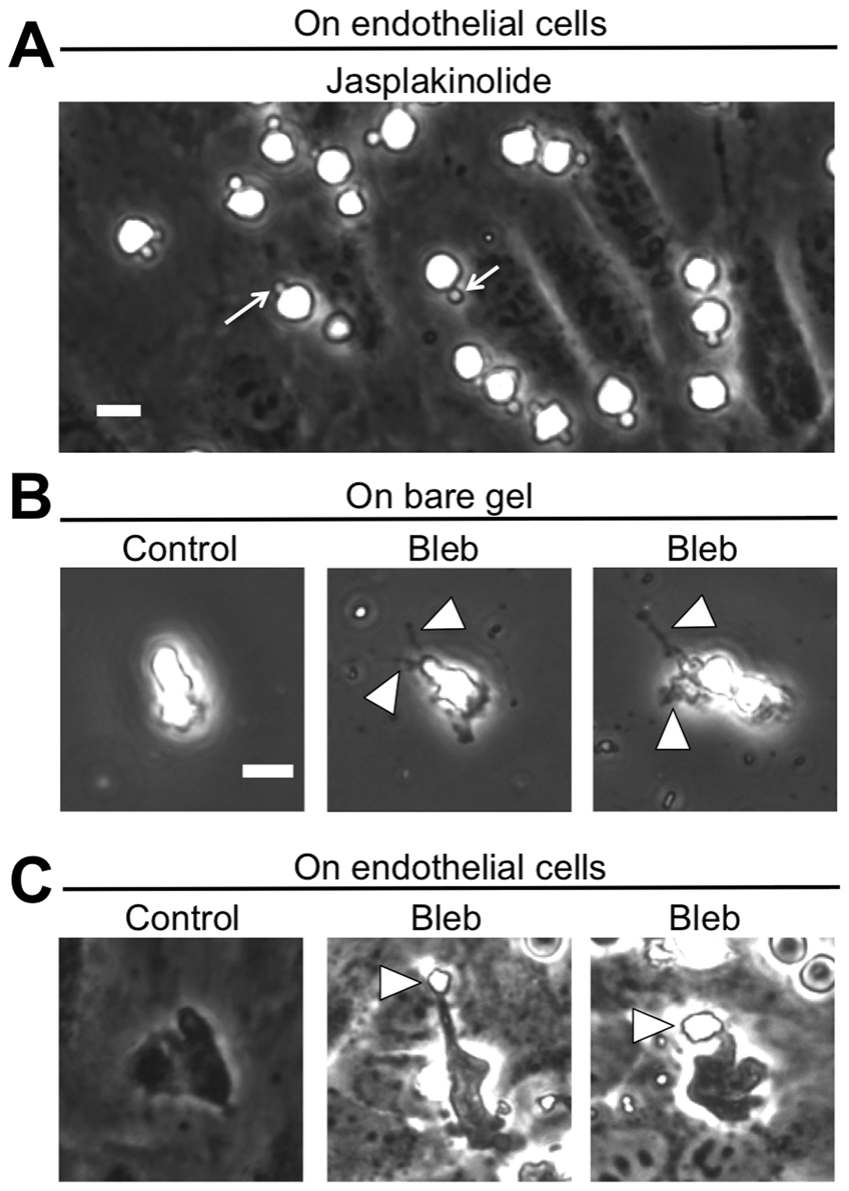
Effects of actin stabilization (via jasplakinolide) or myosin II inhibition (via blebbistatin) on neutrophil morphology. (A) Jasplakinolide-treated neutrophils are shown on a TNF-α-activated HUVEC monolayer on a 5 kPa polyacrylamide gel, approximately 20 minutes after plating neutrophils. White arrows point to protrusion structures that remain (and are not dynamic) throughout the entire timelapse. In subsequent panels, blebbistatin (“bleb”)-treated neutrophils were plated on (B) bare fibronectin-coated polyacrylamide gels (without an endothelium) and activated with 10 nM fMLF or (C) on TNF-α-activated HUVECs on a 5 kPa polyacrylamide gel. Arrowheads in panels B and C point to tails left behind by the neutrophils as it migrates along the gel (panel B) or as it transmigrates through the endothelium (panel C). Two examples of blebbistatin-treated neutrophils are given for panels B and C. Scale bar in panel B is 10 µm and applies to all images in panels A, B, and C.

In comparison with the dimethyl sulfoxide (DMSO) vehicle control, neither nocodazole nor taxol significantly affected the fraction of neutrophils that transmigrated through the endothelium (Fig. 2), suggesting that microtubule dynamics do not contribute to the overall fraction of neutrophils that transmigrate. Meanwhile, treatment of neutrophils with inhibitors of contractility (blebbistatin, ML-7) significantly decreased the fraction of transmigration (Fig. 2; P<0.05). Strikingly, latrunculin-A and jasplakinolide treatments completely abolished the ability of neutrophils to transmigrate (Fig. 2; P<0.001), indicating that neutrophil actin polymerization and depolymerization are essential for neutrophils to cross the endothelial cell barrier. In the case of jasplakinolide treatment, 72±7% of neutrophils retained small protrusions (in comparison with only 11±1% of DMSO-treated neutrophils that remained above the endothelium; P<0.001) after being plated onto the endothelium, and these structures remained throughout the entire 30-minute timelapse, even though no transmigration was observed (Fig. 3A; white arrows).

### Myosin II and MLCK are essential for complete transmigration of the neutrophil “tail”

When plated on bare (endothelium-free) fibronectin-coated polyacrylamide gels, we observed that retraction of the neutrophil's trailing edge (“tail”) was severely impaired in blebbistatin-treated neutrophils, as exhibited by darkened projections that appeared in phase contrast microscopy at the rear of the neutrophil (Fig. 3B; white arrowheads). Meanwhile, on the endothelium, blebbistatin-treated neutrophils also displayed impairment in tail retraction. In this case, the tail appeared as a phase-white piece of the neutrophil, in contrast to the darkened portion of the neutrophil under the endothelium (Fig. 3C). These results indicate the importance of neutrophil myosin II-driven contractility in the neutrophils' ability to completely transmigrate.

**Figure 4:**
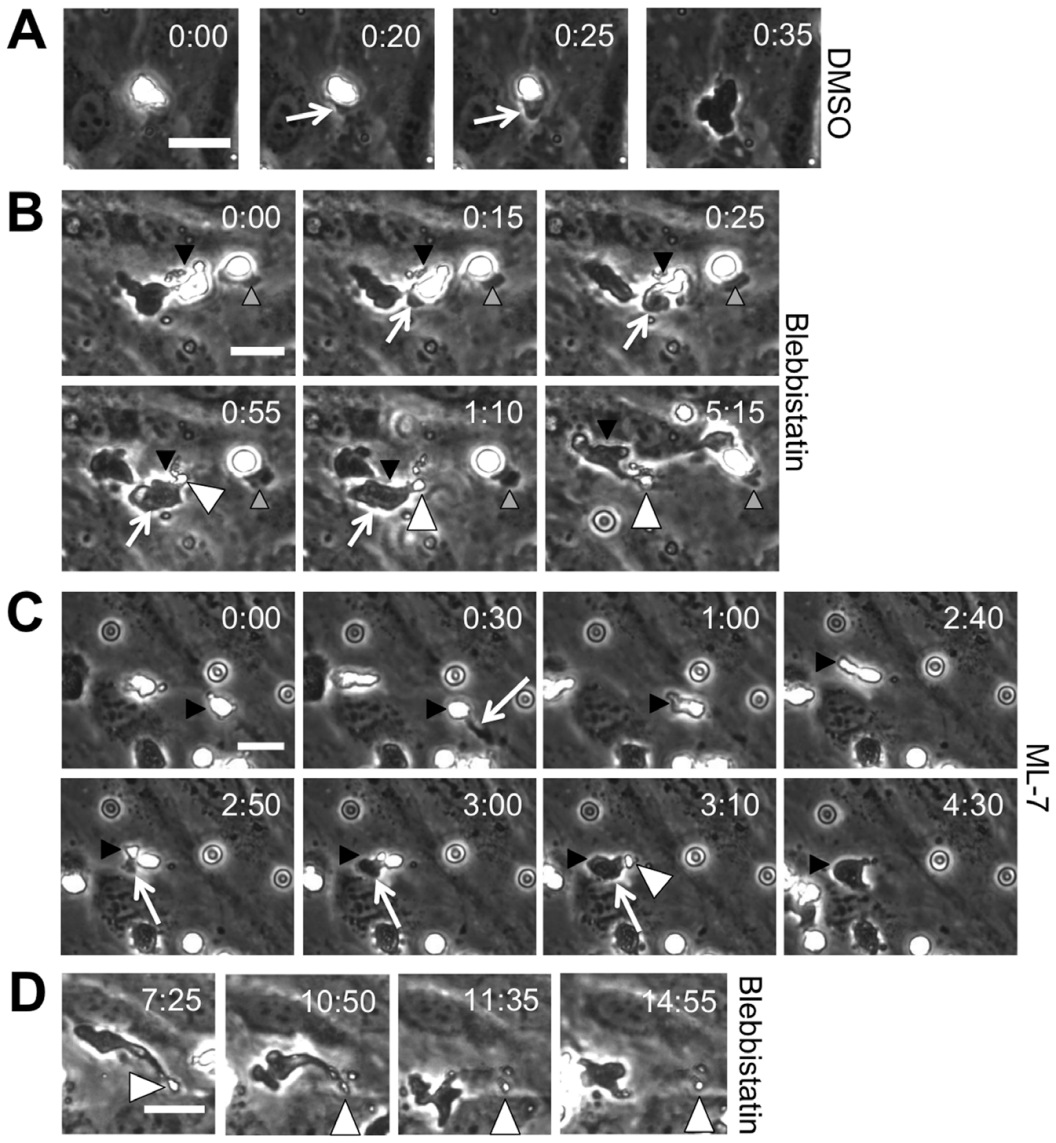
Incomplete transmigration upon inhibition of contractility. (A) A representative phase contrast sequence of a DMSO-treated neutrophil transmigrating through the endothelium on a 5 kPa gel. The neutrophil on top of the endothelium appears bright white in phase contrast microscopy, while the portion that has already transmigrated through the endothelium appears dark (white arrow). (B) A representative phase contrast sequence of blebbistatin-treated neutrophils transmigrating through the endothelium. Black arrowhead points to the first cell of interest. White arrows point to the darkened portion of the neutrophil under the endothelium. White arrowheads point to the portion of the tail that remains above the endothelium for several minutes after the rest of the neutrophil has transmigrated. Gray arrowheads point to another neutrophil that attempts to transmigrate, but does not ever fully succeed. For panels A and B, time = 0:00 (in minutes∶seconds format) in the upper right corner of the first image in each sequence indicates the time of the frame just before transmigration begins. (C) An example of an ML-7-treated neutrophil (indicated by black arrowhead) that makes two attempts at transmigration. A protrusion is sent beneath the endothelium (designated by white arrow at 0:30), but the neutrophil does not transmigrate on this attempt. Later, at time = 2:50 the neutrophil begins to transmigrate again (designated by white arrows in images 2:50–3:10). Similar to blebbistatin, a tail is left behind (white arrowhead at 3:10), but full transmigration, including the tail, occurs by time = 4:30. Scale bar for the first image in each sequence is 20 µm. (D) A time sequence is shown for a blebbistatin-treated neutrophil that transmigrates, leaves a tail behind, and later detaches from the tail. Time after plating neutrophils is shown in the upper right corner of each image in minutes∶seconds fomat. Scale bar in panel D is 20 µm and applies to all images in panel D.

We also observed the dynamics of tail formation via live cell phase contrast imaging during transmigration of blebbistatin- and ML-7-treated neutrophils. When neutrophils were treated with a DMSO vehicle control, they smoothly achieved complete transmigration (Fig. 4A), a process that appeared similar to the untreated case that we have previously published [1]. However, the contractility inhibitors blebbistatin and ML-7 rendered some neutrophils incapable of accomplishing complete transmigration. In these cases, a neutrophil tail remained above the endothelium for several minutes, while the majority of the cell had squeezed through the endothelium (Figs. 4B–C; Movie S2). During this event, the neutrophil often made multiple attempts to begin migration under the endothelium, as it extended protrusions in different directions. However, the tail immobilized above the endothelium prevented the neutrophil from gaining ground under the endothelium. After several minutes, some neutrophils were able to pull the tail through and commence migration under the endothelium (Fig. 4C), while some detached themselves from the tail (Fig. 4D). Several neutrophils were still immobilized by their tail at the end of the 30-minute timelapse experiment.

### Inhibition of myosin II, MLCK, or microtubule dynamics increases time to complete transmigration

**Figure 5:**
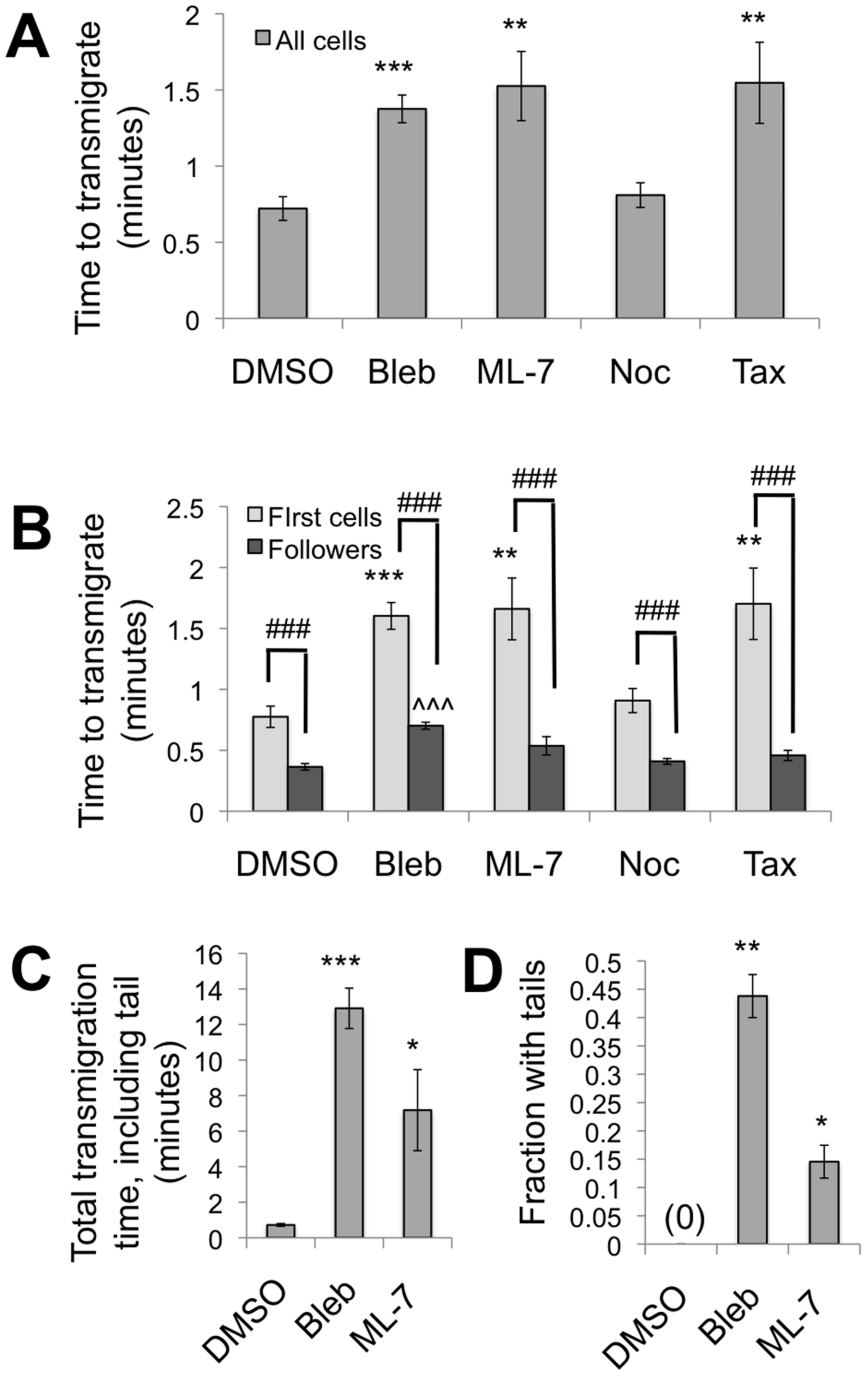
Time to complete transmigration. (A) Transmigration time (through endothelium on 5 kPa gel) for all neutrophils, from when the first protrusion is seen under the endothelium, to complete transmigration (except tails for the cases of blebbistain and ML-7), for all treatments. Bars represent mean ± SEM of pooled data from 3 independent experiments (N = 61, 103, 75, 182, 32 for DMSO, blebbistatin, ML-7, nocodazole, and taxol, respectively). ** indicates P<0.01 and *** indicates P<0.001 with DMSO vehicle control, using a t-test. (B) Transmigration times are shown separately for the “first cells” that enter the endothelium, as well as the “followers” that used the same gap to transmigrate as one of the first cells. Bars represent mean ± SEM of pooled data from 3 independent experiments (For first cells, N = 53, 77, 66, 146, 28, while for followers N = 8, 26, 9, 36, 4 for DMSO, blebbistatin, ML-7, nocodazole, and taxol, respectively). ** indicates P<0.01 and *** indicates P<0.001 with DMSO vehicle control for first cells, while ∧∧∧ indicates P<0.001 with DMSO vehicle control for followers, using a t-test. ### indicates P<0.001 between first cells and followers using a t-test. (C) Total transmigration time, including the time for the final tail to disappear beneath the endothelium. Bars represent mean ± SEM of pooled data from 3 independent experiments (N = 61,68,12 for DMSO, blebbistatin and ML-7, respectively). * and *** indicate P<0.05 or P<0.001 with the DMSO control, using a t-test. (D) Fraction of neutrophils with tails that remain above the endothelium for a prolonged length of time after transmigration of the majority of the neutrophil. Bars represent mean ± SEM of 3–4 independent experiments. * and ** indicate P<0.05 or P<0.01 with the DMSO control, using a t-test.

Our next goal was to evaluate the effects inhibition of myosin II, MLCK, or microtubule dynamics on transmigration time. Here, for the blebbistatin and ML-7 treatments where neutrophils transmigrated and failed to retract the tail (Fig. 4), we considered the time at which the majority of the cell had transmigrated. Overall, blebbistatin (P<0.001), ML-7 (P<0.01), and taxol (P<0.01) nearly doubled the mean time required to complete transmigration (Fig. 5A). Meanwhile, nocodazole had no effect on the transmigration time (Fig. 5A; P>0.05).

In agreement with other reports for monocytes [30], we observed that many neutrophils transmigrated at the same location as a previous neutrophil. Therefore, we next evaluated the time to complete transmigration, considering the “first cells” to cross the endothelium separately from the “followers” that crossed at the same location as another cell. Notably, the followers completed transmigration faster than the first cells, for all treatments (Fig. 5B; P<0.001). Blebbistatin (P<0.001), ML-7 (P<0.01), and taxol (P<0.01) treatments significantly increased the transmigration time for the first cells to cross the endothelium, while only blebbistatin increased the transmigration time for followers (P<0.001) (Fig. 5B). Nocodazole treatment had no effect on transmigration time for either the first cells or the followers (Fig. 5B; P>0.05).

We also quantified the total time required for blebbistatin- and ML-7-treated neutrophils to completely transmigrate, including the tail portion. In these cases, the complete process spanned 13 minutes (P<0.001) and 7 minutes (P<0.05) for blebbistatin and ML-7 treatments, respectively; both were significantly longer than the time for DMSO-treated cells, which was approximately 45 seconds (Fig. 5C). Morphologically, nearly half of the blebbistatin-treated neutrophils (P<0.01) and about 15% of ML-7-treated neutrophils (P<0.05) possessed a tail, in comparison with 0% of DMSO-treated neutrophils (Fig. 5D).

### Transmigration initiation time does not depend on myosin II, MLCK, or microtubules

**Figure 6:**
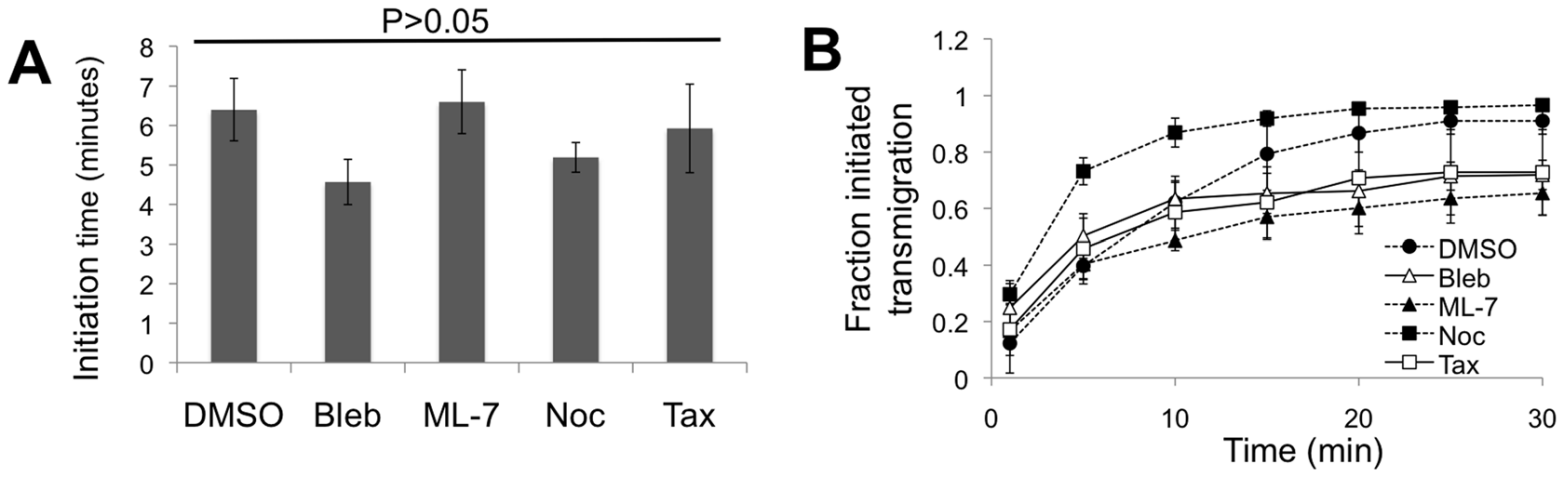
Time to initiate transmigration. (A) Mean time for initiation of transmigration through endothelial cells on a 5 kPa gel. Neutrophils were plated at time = 0. P>0.05 between all treatments and the DMSO vehicle control using a t-test. (B) Fraction of cells that initiated transmigration as a function of time for all treatments. Bars or data points represent mean ± SEM from 3–4 independent experiments.

As a measure of the ability of neutrophils to find a suitable location to transmigrate through the endothelium, we quantified the length of time they spent migrating prior to transmigrating (i.e. transmigration “initiation time”). There was no difference in the mean transmigration initiation time in comparison with the DMSO vehicle control for any of the treatments (Fig. 6A; P>0.05). These results indicate that none of the biophysical perturbations hindered the ability of the neutrophils to find an appropriate transmigration spot. Finally, the curve indicating the fraction of neutrophils that initiated transmigration as a function of time was similar for all treatments, though, as expected, the final saturating fraction varied (Fig. 6B; fraction at 30 minutes also plotted in Fig. 2).

## Discussion

Leukocyte transmigration is a complicated biological event that is critical for the immune response and is also implicated in progression of cardiovascular disease. Previously, we have demonstrated that the mechanical properties of the subendothelial matrix and the physical state of the endothelium are key regulators of the transmigration step of the immune response [1], [6]. Meanwhile, during migration neutrophils sense substrate mechanical properties [21], [22] and also exert measurable traction forces on their substrate [22], [23]. However, little is known about the biophysical molecular machinery active in the neutrophils as they transmigrate. Therefore, in the current work, we hypothesized that neutrophil contractile forces and cytoskeletal dynamics play an active biophysical role during transmigration through endothelial cell-cell junctions.

To address our hypothesis and evaluate the role of neutrophil cytoskeletal dynamics and contractility in trans-endothelial migration, we performed a series of assays in which human neutrophils were treated with pharmacological drugs to target specific biophysical machinery, plated onto a TNF-α-activated endothelium, and imaged during transmigration using phase contrast timelapse microscopy. Our results demonstrate an important role for the neutrophil cytoskeleton in transmigration through endothelial cell-cell junctions. Most notably, F-actin polymerization and depolymerization are essential for transmigration, as evidenced by the lack of transmigration in latrunculin-A- and jasplakinolide-treated neutrophils (Fig. 2). If neutrophil transmigration were simply a passive event in which neutrophils “ooze” through the endothelium, then a reduction in cellular stiffness would likely enhance this event, since leukocytes typically transmigrate through 4–6 µm-sized gaps at EC junctions [10], which is smaller than the 10 µm diameter of an inactivated neutrophil [21]. However, even though inhibition of F-actin polymerization by latrunculin-A reduces cellular stiffness [26], [31], we observed no transmigration in this case, indicating an active role for F-actin polymerization during transmigration. Further, it could be argued that inhibition of F-actin prevents cells from adhering to or migrating along the two-dimensional surface of the endothelium, ultimately precluding them from completing the next step of transmigration. Although some neutrophils transmigrated prior to migration on the endothelium (Fig. 6B), neutrophils treated with latrunculin-A or jasplakinolide did not transmigrate, upon contact with the endothelium; this suggests that actin dynamics are specifically required for both adherence to the endothelium and thus subsequent transmigration.

Meanwhile, microtubule dynamics enable leader neutrophils to cross the endothelium efficiently, since taxol treatment significantly increased the time required to complete transmigration for the first neutrophils at a particular location (Fig. 5B). Taxol treatment may increase transmigration time by reducing neutrophil viscosity [31], thus making it more difficult for the neutrophils to squeeze through the EC junctions. However, microtubule protrusion at the cell's leading edge have also been implicated in the ability of cells to invade into confined spaces [32], which could also explain the role of microtubule dynamics in transmigration. Additionally, it has been shown that microtubule depolymerization (e.g. by nocodazole) causes release of Rho guanine nucleotide exchange factor (GEF) GEF-H1, which activates RhoA [33] and ROCK and therefore enhances cell contractility [34], [35], [36]. Furthermore, microtubule depolymerization causes T-cells to convert from a lamellipodia/uropod-based migratory phenotype to a blebbing-based phenotype through a crosstalk between microtubule dynamics and Rho/ROCK signaling [33]. Thus, we would expect nocodazole treatment to enhance transmigration, given our results that inhibition of contractility reduces (Fig. 2) and slows (Figs. 5B) transmigration. While we did not observe differences in the time to complete transmigration in nocodazole-treated neutrophils (Fig. 5B), we did observe a significant increase in the fraction of transmigration (in comparison with DMSO-treated) at 10 minutes after being plated onto the endothelium (Fig. 6B), and this might be due to enhanced neutrophil contractility after release of GEF-H1 upon microtubule depolymerization. Albeit not statistically significant, it is worth mentioning that the initiation time (or the average time neutrophils spend migrating on the endothelium before transmigration) is shorter for nocodazole-treated neutrophils compared to the DMSO vehicle control (Fig. 6). Together, our experiments point to neutrophil F-actin and microtubule cytoskeletal networks as active contributors to transmigration through endothelial cell-cell junctions.

In addition to cytoskeletal dynamics, we also tested the hypothesis that neutrophil contractility contributes to transmigration through the endothelium. This idea was motivated by our previous work in which inhibition of neutrophil MLCK by treatment with ML-7 reduced the fraction of neutrophils that completed transmigration [1], and here we confirmed those results (Fig. 2). Interestingly, in that previous work, transmigration was reduced by ∼44% when neutrophils were treated with ML-7 on softer subendothelial matrices (0.87 kPa, where ECs are less contractile), in comparison with only a 27% reduction on stiffer subendothelial matrices (280 kPa, where ECs are more contractile) [1]. These results suggest that when the endothelium is less contractile, as on a soft subendothelial matrix, with less (or smaller) intercellular gaps, the neutrophils are required to use their contractile apparatus more in order to squeeze through the tighter endothelial cell-cell junctions. In the current work, we further explored this dynamic role of neutrophil contractility in transmigration.

Work by others has demonstrated that RhoA is necessary for retraction of the trailing edge and complete extravasation in both monocytes [2] and T-cells [37]. *In vivo*, lymphocyte function-associated antigen-1 (LFA-1)-mediated uropod elongation is a final step prior to extravasation of neutrophils, monocytes, and T-cells [38]. RhoA activity and GEF-H1 are both associated with ROCK-mediated contraction of the T-cell uropod during transmigration, as evidenced using a RhoA activity fluorescence resonance energy transfer (FRET) biosensor [37]. RhoA is a small GTPase protein that acts upon Rho-associated protein kinase-1 (ROCK-1) to phosphorylate myosin light chains (MLC) and thus enhance cell contractility. In our work, we built upon these studies by evaluating retraction of the trailing edge of neutrophils treated with blebbistatin or ML-7 to inhibit myosin II and its upstream effector MLCK, respectively. Decreases in the fraction of ML-7- or blebbistatin-treated neutrophils completing transmigration (Fig. 2), increases in total time to complete transmigration (Fig. 5B), and the inability of these neutrophils to completely retract their tails during transmigration (Figs. 3, 4, 5C, and 5D) all support our hypothesis that neutrophil contractility actively contributes to transmigration and demonstrate that myosin II and MLCK are key molecular players in this response.

Intriguingly, our results suggest that the role of the neutrophil's biophysical machinery in transmigration may depend on whether the cell is the first to cross, or whether it crosses at the same location as a previous neutrophil. Consistent with what has been shown in monocytes [10], [30], we observed many neutrophils that transmigrated through the endothelium at the same location as a previous neutrophil. From our work (Fig. 1A) and that of others [10], [30], it is clear that leukocytes induce dislocation and reduction of junctional proteins such as VE-cadherin and augmentation of leukocyte adhesion molecules such as platelet endothelial cell adhesion molecule-1 (PECAM-1) during and after transmigration, and therefore the door, so to speak, is already open for the follower neutrophils, probably providing less resistance. Thus, we hypothesized that follower neutrophils transmigrate faster with less dependence on contractility in comparison with the first cells to cross at a particular location. Indeed, our results supported this hypothesis, showing that for all treatments, follower neutrophils transmigrated 2–3 times faster than the first cells (Fig. 5B). In addition, with the exception of blebbistatin, there was no difference between any of the treatments in the time to complete transmigration for follower cells (Fig. 5B). These results suggest that the first neutrophil utilizes MLCK-mediated contractile forces and microtubule dynamics to transmigrate, but that subsequent neutrophils transmigrating at the same location depend less on MLCK-mediated contractile forces and microtubule dynamics. Thus, the fundamental transmigration mechanism between the two waves of neutrophils may be different.

We would like to alleviate concerns regarding the method of protein inhibition in our experiments. Exogenous gene expression in neutrophils is difficult since they are non-dividing, and successful attempts using nucleofection have resulted in extremely low transfection efficiencies (0.4–1%) [39]; this excludes the possibility of introducing siRNA vectors to knock out proteins. Another possibility would be to use transgenic mice; however, we wished to limit our model system to human neutrophils and ECs, in order to keep our results relevant to human physiology. Therefore, in our assays, neutrophils were treated with pharmacological drugs in suspension after isolation from human blood. Following treatment, neutrophils were washed at least twice in HBSS to ensure the drug did not act on the endothelium and immediately plated onto the endothelium. The majority of neutrophils transmigrated within 1–10 minutes after plating (Fig. 6), which was likely faster than drug washout occurred [40], [41], [42], [43]. Furthermore, staining of neutrophils for α-tubulin, myosin IIA, MLCK, or actin revealed the effectiveness of the various drug treatments even 30 minutes following washout (Fig. S1).

**Figure 7:**
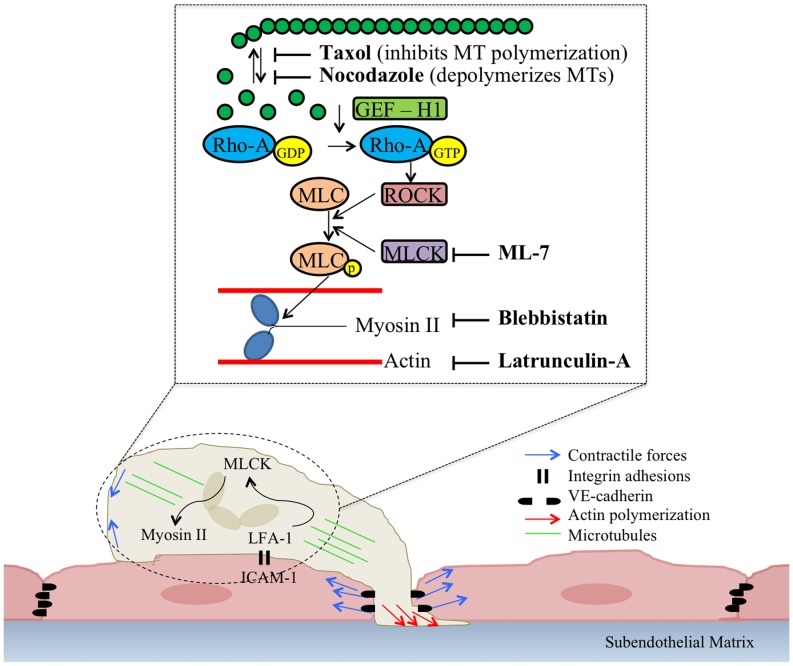
Schematic describing biophysical contributions of neutrophils during transmigration. This schematic incorporates our work, as well as work of others (see text for discussion), and demonstrates the possible cross-talk between the cytoskeleton and contractility-regulating molecules during cell migration. As neutrophil LFA-1 binds to ICAM-1 on the surface of the endothelium, a signaling cascade is initiated inside the ECs, which ultimately leads to EC contraction and gap formation, as well as lateral displacement of VE-cadherin. A signaling cascade is simultaneously initiated in the neutrophils. Neutrophil actin polymerization initiates transmigration through the endothelial cell-cell junctions by protruding at the leading edge. Microtubule depolymerization activates GEF-H1, which stimulates the exchange of GDP to GTP on RhoA, and subsequent signaling to ROCK. Both ROCK and MLCK phosphorylate MLC, which induces actin-myosin contraction. MLCK- and myosin II-mediated contractile forces at the uropod promote retraction at the trailing edge, and by inhibiting these forces, the neutrophils cannot fully complete transmigration.

The schematic in Figure 7 demonstrates how our work might fit into the context of what is known about the cross-talk between the cytoskeleton and contractility-regulating molecules during cell migration. As neutrophil LFA-1 binds to ICAM-1 on the surface of the endothelium, a signaling cascade is initiated inside the ECs, which ultimately leads to EC contraction and gap formation, as well as lateral displacement of VE-cadherin [1], [10]. However, our data supports the hypothesis that a signaling cascade is simultaneously initiated in the neutrophils. Actin polymerization in the neutrophils commences transmigration through the endothelial cell-cell junctions by protruding at the leading edge. Microtubule dynamics (specifically, depolymerization) activate GEF-H1, which stimulates the exchange of GDP to GTP on RhoA, and subsequent signaling to ROCK [34], [35], [36]. Both ROCK [44] and MLCK [3], [4] phosphorylate MLC, which induces actin-myosin contraction. MLCK- and myosin II-mediated contractile forces at the uropod promote retraction at the trailing edge, and by inhibiting these forces, the neutrophils cannot fully complete transmigration.

In summary, we have provided significant data in support of our hypothesis that neutrophil contractile forces and cytoskeletal dynamics play an active biophysical role during transmigration through endothelial cell-cell junctions. Our results support a model (Fig. 7) whereby transmigration involves neutrophil F-actin polymerization, microtubule dynamics, and MLCK- and myosin II-dependent contractility, in addition to the role of EC contractility that we have previously demonstrated [1], [6]. We observed that >93% of neutrophils exploit the paracellular mode of transmigration in our *in vitro* model, and neutrophils do not alter their preferential pathway based on the state of contractility in the endothelium. Next, we showed that both neutrophil F-actin polymerization and microtubule dynamics play an active role in neutrophil transmigration. Further, inhibition of myosin II- and MLCK-mediated neutrophil contractile forces renders neutrophils incapable of fully retracting their trailing edge under the endothelium for several minutes after the majority of the neutrophil has transmigrated. We also demonstrated that inhibition of neutrophil contractile forces and stabilization of microtubules doubled the time to complete transmigration for the first neutrophils to cross the endothelium. Notably, the time to complete transmigration is significantly reduced for subsequent neutrophils that cross at the same location as a previous neutrophil, and this time is less dependent on neutrophil contractile forces and microtubule dynamics. These results suggest that the first neutrophil “opens the door” in the endothelium VE-cadherin, making it easier for subsequent neutrophils to pass through the same gap. While this may be an advantage for the immune response, the gaps in the endothelium created by leukocytes could also have adverse consequences, possibly serving as entry points for tumor cells to extravasate from (or intravasate into) the blood stream to a nearby tissue, thus accelerating the metastatic process. Collectively, this work demonstrates that neutrophils play an active biophysical role in the transmigration step of the immune response. For further discussion, please see supplemental discussion section.

## Supporting Information

Figure S1 **Neutrophils were stained for target proteins (red) before (“pre-drug”), immediately after (“post-drug”), or after washout (“washout”) of treatment with various pharmacological drugs.** Nuclei were stained by Hoechst and appear blue. Scale bar in lower right image is 10 µm and applies to all images.
(TIF)

Movie S1 **Phase contrast (left), VE-cadherin-GFP (middle), and overlay (right) movies of neutrophils migrating through a TNF-α-activated HUVEC monolayer.** White circle in VE-cadherin-GFP (middle) movie in the first frame indicates the location at which a neutrophil transmigrates at a tricellular junction, creating a gap in the endothelial cell VE-cadherin, through which several “follower” neutrophils also transmigrate before the VE-cadherin translocates back to the junction. Scale bar is 10 µm. Time in upper right corner is in minutes∶seconds format, where time = 0 corresponds to the time within one minute of plating neutrophils onto the endothelium.
(AVI)

Movie S2 **Phase contrast movie of blebbistatin-treated (15 µM) neutrophils transmigrating through a TNF-α-activated HUVEC monolayer.** Several neutrophils here leave behind a phase-white “tail” after the main portion of the neutrophil has transmigrated. Scale bar is 20 µm. Time in upper left corner is in minutes∶seconds format, where time = 0 corresponds to the time within one minute of plating neutrophils onto the endothelium.
(AVI)

Text S1 (PDF)
