# Supplementary figures and images for: Human Neutrophil Cytoskeletal Dynamics and Contractility Actively Contribute to Trans-Endothelial Migration
Source: PLoS One. 2013 Apr 23;8(4):e61377. doi: 10.1371/journal.pone.0061377 (PMC3634075; doi:10.1371/journal.pone.0061377)

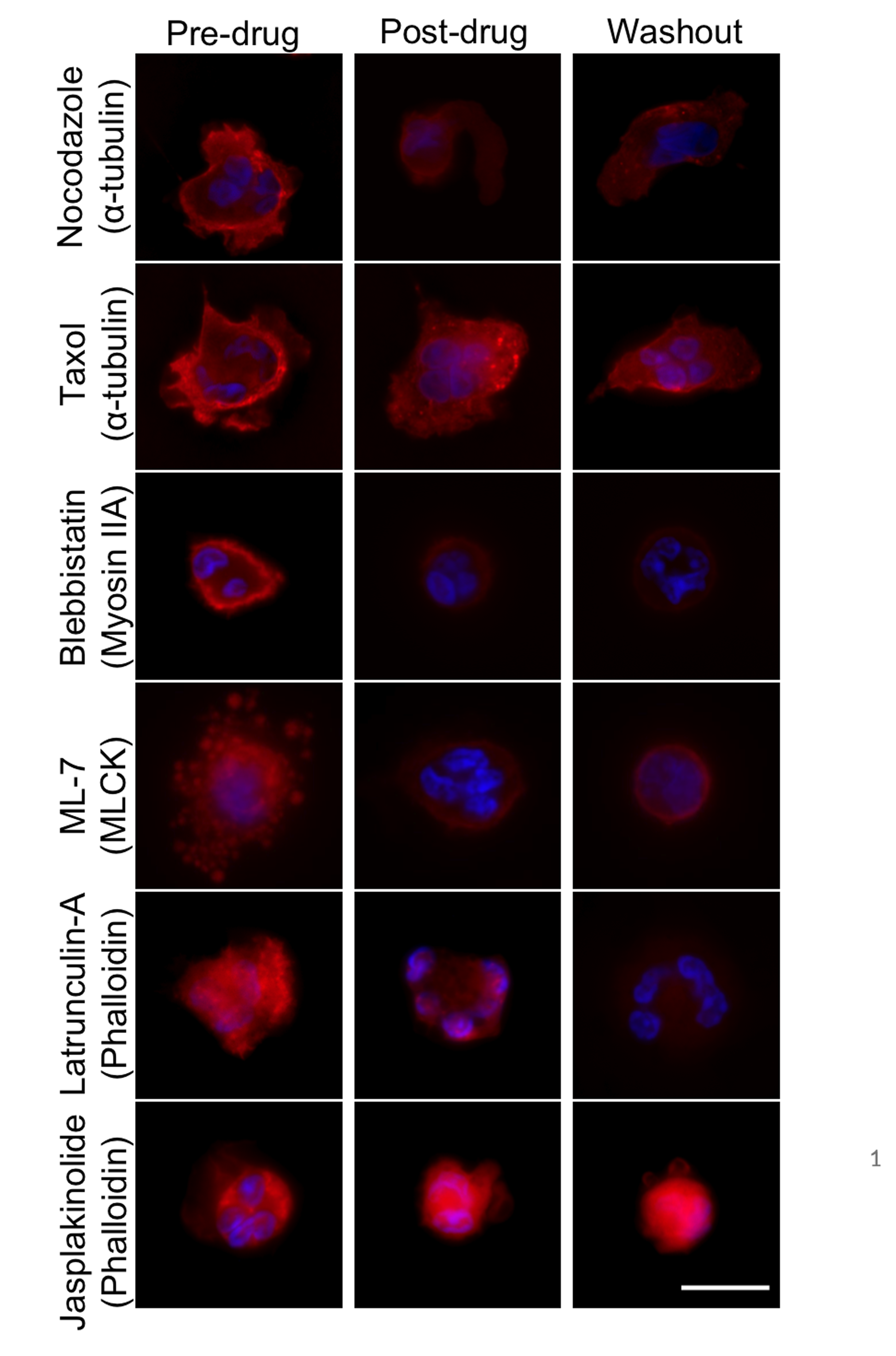

Supplement: Figure S1 — Neutrophils were stained for target proteins (red) before (“pre-drug”), immediately after (“post-drug”), or after washout (“washout”) of treatment with various pharmacological drugs. Nuclei were stained by Hoechst and appear blue. Scale bar in lower right image is 10 µm and applies to all images. (TIF) [file pone.0061377.s001.tif]
