# Supplementary material for: Human Neutrophil Cytoskeletal Dynamics and Contractility Actively Contribute to Trans-Endothelial Migration
Source: PLoS One. 2013 Apr 23;8(4):e61377. doi: 10.1371/journal.pone.0061377 (PMC3634075; doi:10.1371/journal.pone.0061377)
Supplement: Text S1 — (PDF) [file pone.0061377.s004.pdf]

Supplemental Information for

Human neutrophil cytoskeletal dynamics and contractility actively contribute to trans-endothelial migration

Kimberly M. Stroka, Heather N. Hayenga, and Helim Aranda-Espinoza*

Fischell Department of Bioengineering, University of Maryland, College Park, Maryland, United States of America

---

Supplemental Discussion

Route of transmigration

Our first goal was to establish the predominant route by which neutrophils transmigrate in our experimental system, since it is possible that neutrophils use different molecular machinery to transmigrate by the paracellular and transcellular routes. By infecting the endothelium with AdV-VE-cadherin-GFP to label cellular borders and imaging live neutrophil transmigration, we established that the paracellular route (between cell-cell junctions) is highly preferable (>93% of cases) for neutrophil transmigration (Fig. 1B). This result agrees with previous reports for lymphocyte transmigration through HUVECs, where the paracellular route accounts for ~90% of the overall transmigration events [1]. Also similar to lymphocytes [1], the time to complete transmigration for neutrophils does not differ between the paracellular and transcellular routes (Fig. 1C), though neutrophils complete transmigration about six times faster than lymphocytes, likely due to the neutrophils' role as the immune system's first responders to injury or infection. Further, it is known that neutrophil and lymphocyte extravasation and vascular permeability in the cremaster, lung, and skin are completely inhibited by selectively blocking the paracellular route *in vivo* through stabilization of the VE-cadherin-catenin complex,

indicating that in these tissues, leukocytes do not switch to the transcellular route when the paracellular route is blocked [2]. Our results agree with this idea, since varying subendothelial matrix stiffness does not change the route by which neutrophils transmigrate; for example, on softer substrates, where junctions are "stabilized" we do not observe more transcellular transmigration (Fig. 1B). Thus, in the first set of experiments, we confirmed that neutrophil transmigration is mostly paracellular and is independent of subendothelial matrix stiffness in our *in vitro* system and therefore a representative subendothelial matrix stiffness of a "healthy" blood vessel (5 kpa) could be used in subsequent experiments to test the biophysical role of neutrophils in transmigration.

Neutrophil migration prior to transmigration

It should also be mentioned that while we did not specifically quantify neutrophil migration along the endothelium (prior to transmigration), neutrophils were motile for all treatments, with the exception of latrunculin-A (where cells were immobilized due to the treatment). On average, cells spent the same amount of time migrating prior to transmigrating (Fig. 6A), which is an indication that they had equal time to explore the endothelium. Thus, we chose to focus on the event of transmigration. However, microtubules and contractility are both known to play important roles in neutrophil motility along two-dimensional substrates, and thus future work could analyze their role in migration along the endothelium.

Relevance to myosin II activity on 2D substrates

Our results are also supported by previous work on the localization and activity of myosin II during migration on two-dimensional flat substrates. For example, it has been shown

that phosphorylated myosin II and RhoA accumulate at the rear of HL60 cells during chemotaxis on two-dimensional flat surfaces, and that inhibition of myosin II by blebbistatin impairs rear retraction [3]. Furthermore, Ca^{2+}-dependent myosin II activation is required for neutrophil uropod retraction during migration along fibronectin-coated substrates [4]. While the aforementioned studies have been completed in the absence of an endothelium, other work has shown that adhesion to and migration across the pulmonary endothelium are impaired in MLCK-/- neutrophils [5]; impairment of adhesion and migration was attributed not to phosphorylation of myosin II, but rather to defects in MLCK-mediated activation of β2 integrins [5]. Herein we observed that a few neutrophils failed to adhere and migrate on the endothelium either with blebbistatin or ML-7, indicating that disruption of actomyosin contractility plays a role in both adhesion and transmigration.

Effects of drugs on other signaling pathways

Drug concentrations were chosen based on their ability to specifically target the desired protein or process with little or no impact on other signaling pathways, according to previous work on a variety of cell types. For example, blebbistatin inhibits most nonmuscle myosin II above 0.5 to 5 μM without inhibiting other myosins [6]. ML-7 has been shown to be an effective and specific inhibitor of MLCK at concentrations in the range 10-20 μM [7,8]. Inhibition of actin dynamics has also been shown to occur by 1 μM latrunculin-A [9] or jasplakinolide [10]. Furthermore, others have quantified a significant decrease in microtubule content after addition a similar treatment with nocodazole, as well as a slight, yet not significant, increase in microtubule content after taxol incubation [11]. In the main text we raise the point that modification of

microtubule dynamics (by nocodazole or taxol) may interfere with Rho GTPase signaling, which could ultimately explain our observed effects on transmigration dynamics.

Supplemental References

1. Carman CV, Sage PT, Sciuto TE, de la Fuente MA, Geha RS, et al. (2007) Transcellular diapedesis is initiated by invasive podosomes. Immunity 26: 784-797.

2. Schulte D, Kuppers V, Dartsch N, Broermann A, Li H, et al. (2011) Stabilizing the VE-cadherin-catenin complex blocks leukocyte extravasation and vascular permeability. Embo Journal 30: 4157-4170.

3. Xu J, Wang F, Van Keymeulen A, Herzmark P, Straight A, et al. (2003) Divergent signals and cytoskeletal assemblies regulate self-organizing polarity in neutrophils. Cell 114: 201-214.

4. Eddy RJ, Pierini LM, Matsumura F, Maxfield FR (2000) Ca2+-dependent myosin II activation is required for uropod retraction during neutrophil migration. J Cell Sci 113 (Pt 7): 1287-1298.

5. Xu J, Gao XP, Ramchandran R, Zhao YY, Vogel SM, et al. (2008) Nonmuscle myosin light-chain kinase mediates neutrophil transmigration in sepsis-induced lung inflammation by activating beta2 integrins. Nat Immunol 9: 880-886.

6. Limouze J, Straight AF, Mitchison T, Sellers JR (2004) Specificity of blebbistatin, an inhibitor of myosin II. J Muscle Res Cell Motil 25: 337-341.

7. Bain J, McLauchlan H, Elliott M, Cohen P (2003) The specificities of protein kinase inhibitors: an update. Biochem J 371: 199-204.

8. Mansfield PJ, Shayman JA, Boxer LA (2000) Regulation of polymorphonuclear leukocyte phagocytosis by myosin light chain kinase after activation of mitogen-activated protein kinase. Blood 95: 2407-2412.

9. Coue M, Brenner SL, Spector I, Korn ED (1987) Inhibition of actin polymerization by latrunculin A. FEBS Lett 213: 316-318.

10. Holzinger A (2001) Jasplakinolide. An actin-specific reagent that promotes actin polymerization. Methods Mol Biol 161: 109-120.

11. Kraning-Rush CM, Carey SP, Califano JP, Smith BN, Reinhart-King CA (2011) The role of the cytoskeleton in cellular force generation in 2D and 3D environments. Phys Biol 8: 015009.
